# Effect of Integrating Access to a Prescription Drug Monitoring Program Within the Electronic Health Record on the Frequency of Queries by Primary Care Clinicians: A Cluster Randomized Clinical Trial

**DOI:** 10.1001/jamahealthforum.2022.1852

**Published:** 2022-06-05

**Authors:** Hannah T. Neprash, David M. Vock, Alexandra Hanson, Brent Elert, Sonja Short, Pinar Karaca-Mandic, Alexander J. Rothman, Genevieve B. Melton, David Satin, Rebecca Markowitz, Ezra Golberstein

**Affiliations:** 1 Division of Health Policy and Management, School of Public Health, University of Minnesota, Minneapolis; 2 Division of Biostatistics, School of Public Health, University of Minnesota, Minneapolis; 3 Fairview Health Services, Minneapolis, Minnesota; 4 Institute for Health Informatics, University of Minnesota, Minneapolis; 5 Carlson School of Management, University of Minnesota, Minneapolis; 6 Department of Psychology, College of Liberal Arts, University of Minnesota, Minneapolis; 7 Department of Surgery, Medical School, University of Minnesota, Minneapolis; 8 Center for Learning Health System Sciences, University of Minnesota, Minneapolis; 9 Department of Family Medicine and Community Health, Medical School, University of Minnesota, Minneapolis; 10 Department of Medicine, Medical School, University of Minnesota, Minneapolis

## Abstract

**Importance:**

Tools that are directly integrated with the electronic health record (EHR) workflow can reduce the hassle cost of certain guideline-concordant practices, such as querying a prescription drug monitoring program (PDMP) before prescribing opioids.

**Objective:**

To investigate the effect of integrating access to a PDMP within the EHR on the frequency of program queries by primary care clinicians.

**Design, Settings, and Participants:**

The PRINCE (Prescribing Interventions for Chronic Pain Using the Electronic Health Record) randomized trial used a factorial cluster design at the clinic level in 43 primary care clinics in Minnesota. In all, 309 clinicians participated; 161 clinicians were given EHR-integrated access to PDMP at the intervention clinics, and 148 clinicians had the usual access at the control clinics. The intervention went live on August 27, 2020, and data were collected through March 3, 2021.

**Intervention:**

Single sign-on access to the Minnesota PDMP was integrated into the EHR, allowing clinicians to query a patient’s controlled substance prescription and dispensing history as recorded in the Minnesota PDMP directly from the patient’s EHR record without logging into a separate web portal. Additionally, the integration tool alerted clinicians and reminded them to review the PDMP if a patient had 3 or more opioid prescriptions in the past year and 1 or more in the past 6 months. Clinics in the control group did not receive access to the EHR-integrated PDMP tool; instead, these participants logged into the PDMP web portal separately.

**Main Outcomes and Measures:**

Monthly PDMP query counts for primary care clinicians, overall and by modality (EHR-based, web-based, via a clinical delegate), adjusted for clinician characteristics, including type (physician, nurse practitioner, physician assistant), sex, and years in practice. Data were analyzed from August 2021 to May 2022.

**Results:**

Of the 43 participating clinics with 309 clinicians, 21 clinics with 161 clinicians (102 [63.4%] women; 114 [70.8%] physicians; tenure, 10.6 [4.4] years) received the PDMP integration intervention. Baseline unadjusted monthly PDMP query rates for the average clinician were 6.6 (95% CI, 4.4-9.9) vs 8.8 (95% CI, 6.0-13.1) queries in the control vs the PDMP integration group, respectively. During the intervention, PDMP query rates for the average clinician were 6.9 (95% CI, 4.7-10.3) vs 14.8 (95% CI, 10.0-22.0) queries among the control vs the PDMP integration group, respectively. Compared with the control group, the EHR-integrated PDMP tool produced a 60% greater increase in the relative change in monthly PDMP queries (95% CI, 51%-70%). An increase in PDMP queries via the EHR-integrated PDMP tool drove this increase, while web-based and delegate queries declined by 39% more among the intervention compared with the control group (95% CI, 34%-43%).

**Conclusions and Relevance:**

This cluster randomized clinical trial found that integrating access to the PDMP in the EHR increased PDMP-querying rates, suggesting that direct access reduced hassle costs and can dramatically improve adherence to guideline-concordant care practices among primary care clinicians.

**Trial Registration:**

ClinicalTrials.gov Identifier: NCT04601506

## Key Points

**Question:**

Does direct access to the prescription drug monitoring program (PDMP) from within the electronic health record (EHR) increase the frequency of PDMP queries by primary care clinicians?

**Finding:**

This cluster randomized clinical trial involving 309 clinicians in 43 primary care clinics found that providing direct access to a PDMP tool from the EHR increased PDMP queries by 60% compared with clinicians in control clinics who did not have EHR-integrated access.

**Meaning:**

The findings of this trial demonstrate that direct access from the EHR to a PDMP can increase the provision of guideline-concordant care.

## Teaser

This randomized clinical cluster trial of more than 40 clinics and 300 clinicians investigates whether providing access to a prescription drug monitoring program from within the electronic health record increases querying frequency.

## Introduction

Modern primary care involves many activities beyond face-to-face patient care. Clinicians must document the care they provide—an activity that requires the same amount of time as the actual patient interaction.^1^ Clinicians also place orders for follow-up care, secure prior authorization for certain procedures, and respond to patients’ questions through messaging portals—and these additional administrative activities may be associated with clinician burnout.^2,3^ Although many administrative activities are important for patient care, identifying ways to make administrative activities more efficient is crucial to optimizing the time that clinicians spend with patients and promoting clinician well-being.

An example of a new demand being placed on clinicians’ time is the guideline-recommended practice of consulting a prescription drug monitoring program database (PDMP) before prescribing any controlled substance, such as an opioid medication. In response to the opioid epidemic, nearly every state has established a PDMP since 2005, and the US Centers for Disease Control and Prevention has recommended that clinicians use it to review patient history of controlled substance prescriptions.^4,5^ The PDMPs track all controlled substance prescription fills and are intended to provide clinicians with patient prescribing history. Initially, clinicians were not required to consult the state PDMP before prescribing an opioid or controlled substance; therefore, use was low and PDMPs were not found to have any effect on opioid prescribing.^6,7^ Subsequent state-level mandates requiring clinicians to query the PDMP before prescribing an opioid medication have modestly reduced opioid prescribing and opioid deaths.^7,8,9^

Despite the documented effect of PDMP mandates, PDMP querying rates are far from 100%. A single-state study found that prescribers queried the PDMP for only 52% of covered opioid prescriptions, despite a state mandate.^10^ Low rates of PDMP querying reflect a confluence of factors ranging from resentment of legislative incursion into clinical decision-making, to poor usability of PDMPs associated with a lack of integration with electronic health records (EHRs) and the time-consuming nature of information retrieval.^3,11,12,13^ Although the technology to integrate a PDMP directly into EHRs exists, implementation of this functionality remains low, with only 1 in 10 hospitals currently using it.^3^ To our knowledge, there is no available evidence on integration of PDMP access into the EHR in an ambulatory care setting.

To encourage clinicians to query the PDMP before prescribing opioids, we randomized access to direct EHR-integration of Minnesota’s PDMP. On January 1, 2021, clinicians in Minnesota were legislatively mandated to query the PDMP before any initial prescription for schedule II through IV opioids.^14^ This PDMP integration occurred as part of the PRINCE (Prescribing Interventions for Chronic Pain Using the Electronic Health Record) trial—which tested behavioral nudges designed to improve pain treatment and opioid prescribing appropriateness. In this article, we report on how access to the PDMP integration affected the number of PDMP queries. Although the PDMP integration was only 1 of 2 treatments randomized in the PRINCE trial (the other treatment being a choice architecture intervention described elsewhere^15^), we focus exclusively on it in this article because clear evidence of effectiveness led to the early expansion of the PDMP integration throughout all clinics participating in the PRINCE trial.

## Methods

This study followed the Consolidated Standards of Reporting Trials (CONSORT) reporting guidelines. All study procedures were reviewed and approved by the University of Minnesota institutional review board prior to study commencement, with a waiver of informed consent for participating clinicians and patients, per the trial protocol (Supplement 1).

### Study Design and Participants

The PRINCE trial involved 43 primary care clinics owned or affiliated with the academic health system, M Health Fairview, based in Minnesota. These clinics used the Epic EHR system (Epic Systems Corp) and represent all the primary care clinics in this system except 4 that were excluded because they had already implemented the intervention.

Clinicians were identified as potential study participants if they were a primary care clinician (physician, physician assistant, nurse practitioner) working in a study clinic and were not a first-year resident (ie, without prescriptive authority). All potential study participants were invited to give consent to allow access to their PDMP-querying data from the Minnesota Board of Pharmacy, and that consent was obtained electronically approximately 8 weeks after the start of the trial.

The PRINCE trial used a 2 × 2 factorial design to test the EHR-integrated PDMP tool and a choice architecture intervention. The 43 clinics were randomized 1:1:1:1 to the following arms: (1) care as usual control; (2) the choice architecture intervention^15^; (3) access to the EHR-integrated PDMP tool; and (4) the choice architecture and integrated PDMP access. For the purposes of this study, PDMP integration referred to clinics in arms 3 and 4, while remaining clinics were considered to be in the “no PDMP integration” control group. The randomization and allocation of clinics to interventions was conducted by the study biostatistician (D.M.V.). Randomization was at the clinic level to avoid concerns of spillover effects across clinicians within clinics and to be feasible in this clinical setting. Randomization was constrained to avoid imbalances in the number of clinics in each system branch (Fairview, HealthEast, University of Minnesota Physicians, independent Fairview affiliate) randomized to each condition. Randomization was not constrained so that the number of clinicians in each system branch or other clinician characteristics were balanced among the groups.

Clinicians in the PDMP-integration clinics received access to the Appriss PMP Gateway (Bamboo Health) tool. This proprietary tool uses a single sign-on, allowing clinicians to query a patient’s controlled substance prescription and dispensing history as recorded in the Minnesota PDMP directly from the patient’s record in Epic, rather than logging into a separate PDMP web portal outside of Epic. The tool presents information about the history of opioids, sedatives, and stimulants prescribed to the patient and by prescriber, along with a calculated overdose risk score. An Epic alert reminded clinicians that the PDMP needed review if their current patient had 3 or more opioid prescriptions in the past year and 1 or more in the past 6 months. Additional details on the Appriss PMP Gateway tool are available in Supplement 1.

The Appriss PDMP Gateway tool was a considerable change from practice as usual. Previously (and for clinics not receiving this intervention), clinicians had to separately log into the Minnesota PDMP website (outside of Epic) to query the PDMP. Each query required the clinician or their delegate to use login credentials specific to the Minnesota PDMP.

Data on clinicians’ PDMP-querying behavior came from the Minnesota Board of Pharmacy, with observations at the clinician-month level. Outcomes of interest included the monthly count of all PDMP queries (at the clinician level), and query count by modality (via the web-based PDMP portal, via the EHR-integrated PDMP tool, or by a registered clinical delegate via the web portal) for the 13 months prior to the start of the intervention and the 6 months following initiation.

**Figure 1.**
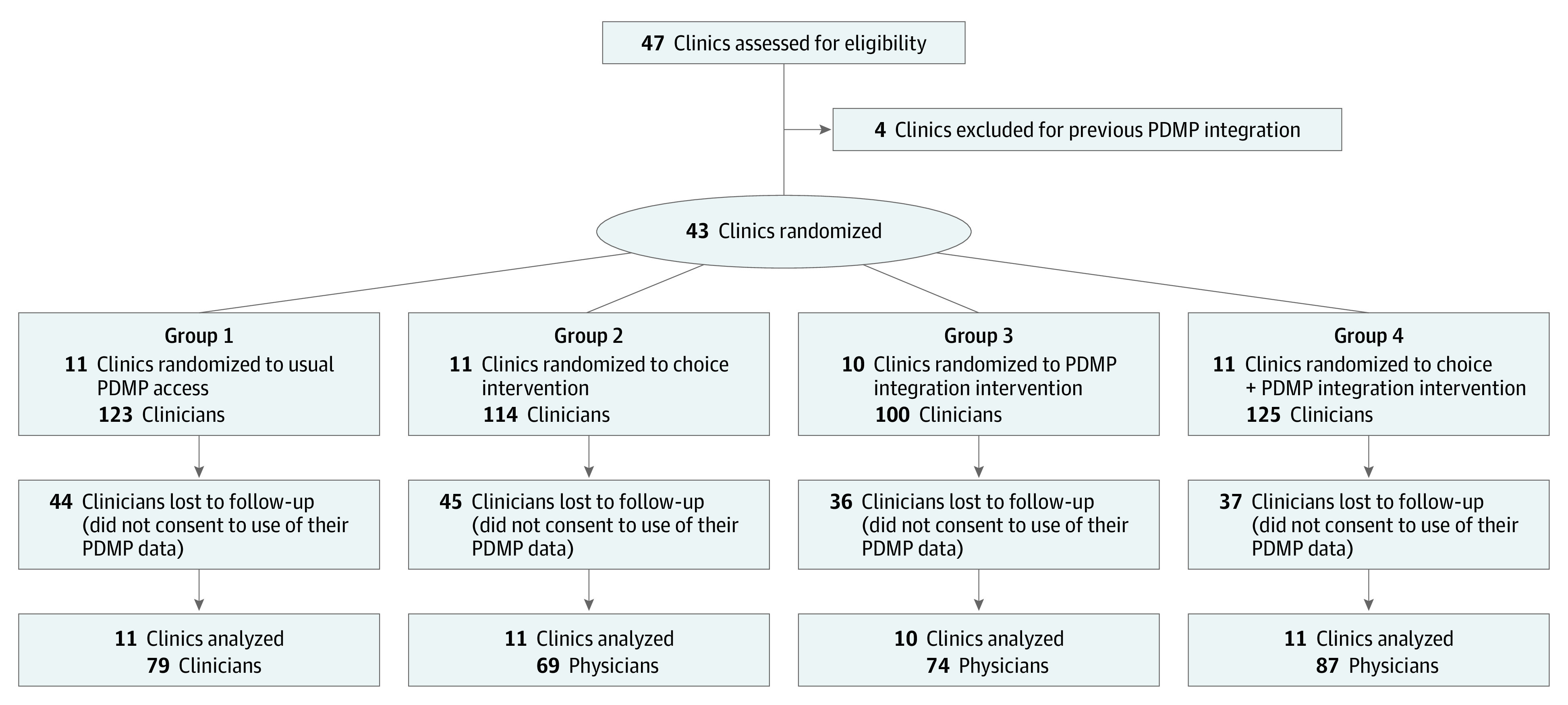
CONSORT Flow Diagram

Intervention assignment occurred at the clinic level, with 21 clinics receiving the PDMP integration intervention. This intervention went live in 17 clinics in the PDMP integration group on August 27, 2020, and in the remaining 4 clinics on September 15, 2020 (Figure 1). While initially intended to last 12 months, the PDMP integration was made available to all clinics within M Health Fairview beginning on March 3, 2021, per the recommendation of the independent data monitoring committee after an interim data review. This study relied on data from the period immediately prior to the Fairview-wide availability of the EHR-integrated PDMP tool. Data on clinician characteristics came from the National Plan & Provider Enumeration System, and included clinician sex, years in practice, and degree. Blinding was not possible at the clinic and clinician level in this pragmatic trial. Except for the study biostatistician (D.M.V.) who implemented the interim data review and the project manager (A.H.) who coordinated the intervention implementation, the study team was blinded to the intervention assignments.

### Statistical Analysis

We compared clinician characteristics across clinics that did and did not receive the EHR-integrated PDMP intervention. To quantify the effect of the EHR-integrated PDMP intervention, we fit Poisson mixed-effect regression models for the PDMP query count each month for each clinician using a log link. We included an indicator for baseline vs intervention period, an interaction between study group and time period, clinician characteristics (sex, years of practice, degree), and health system branch (Fairview, HealthEast, University of Minnesota Physicians, independent Fairview affiliate) as fixed effects. All models included practice and clinician-within-practice nested random effects. The interaction between study group and time period captures the difference on the log scale of the mean change from baseline to the intervention period between the 2 groups (ie, a difference-in-differences regression on the log scale). Exponentiating this coefficient yielded the ratio of the relative change (intervention to baseline period) in mean monthly query count between the PDMP integration and control conditions; values greater than 1.0 indicate that clinicians in the PDMP integration condition had a greater relative increase in the mean monthly number of PDMP queries compared with the control. We also fit similar models without the clinician and clinic fixed effects. By setting the random effect equal to zero in this fitted model, we estimated the average number of queries for an average clinician in an average clinic, both before and after the intervention period.

Models were fit by maximizing the residual, subject-specific (pseudo) likelihood using SAS proc glimmix (SAS Institute Inc). To allow for overdispersion, we included a random residual component; we used (pseudo)-likelihood-based standard errors to quantify uncertainty and construct Wald-type 95% CIs. We fit models to the overall cohort as well as by subgroups defined by clinician type (physician and nurse practitioner/physician assistant) and median years of practice (<13 or ≥13 years). As a sensitivity analysis, we also fit similar models with intervention as a 4-level variable, allowing the assessment of the effect of the choice architecture intervention on PDMP queries.

Statistical tests were 2-tailed and *P* values < .05 were considered statistically significant. Data analyses were performed from August 2021 to May 2022 using Stata, release 16.1 (StataCorp LLC), and SAS, version 9.4 (SAS Institute Inc).

## Results

The PRINCE study included a total of 472 clinicians from 43 participating primary care clinics; 309 clinicians (65.4%; 189 [61.2%] women; 120 [38.8%] men; data on age and race/ethnicity were not collected) consented to have their PDMP query counts accessed through the Minnesota Board of Pharmacy (Figure 1). Rates of consent to PDMP data access did not vary significantly across the randomized groups (χ^2^ [PDMP integration], 2.65; *P* = .10). The PDMP integration group comprised 161 participants (102 [63.4%] women; 59 [36.7%] men; 114 [70.8%] physicians; tenure, 10.6 [4.4] years).

Table 1 summarizes the baseline characteristics of the 309 consenting clinicians by whether they practiced in a clinic that received the PDMP integration. Clinician characteristics varied little by randomized group, with physicians representing the majority of clinicians in the PDMP integration and control clinics (70.8% vs 72.3%; *P* = .77). Clinician sex and years in practice were similar across PDMP integration and control clinics. However, clinicians in the PDMP integration group were more likely to practice at clinics affiliated with Fairview Health System, rather than directly owned by it (21.1% vs 5.4%; *P* < .01).

**Table 1.** Baseline Characteristics of 309 Participating Clinicians, by Study Group

Characteristic	Total, No. (%)	PDMP integration intervention, No. (%)		*P* value
		No	Yes	
Clinician				
Clinician type				
Nurse practitioner	50 (16.2)	21 (14.2)	29 (18.0)	.36
Physician assistant	38 (12.3)	20 (13.5)	18 (11.2)	.53
Physician	221 (71.5)	107 (72.3)	114 (70.8)	.77
Sex				
Female	189 (61.2)	87 (58.8)	102 (63.4)	.41
Male	120 (38.8)	61 (41.2)	59 (36.7)	.41
Years since NPI assigned, mean (SD)	10.5 (4.3)	10.4 (4.3)	10.6 (4.4)	.66
Clinic health system				
Fairview	150 (48.5)	78 (52.7)	72 (44.7)	.16
Fairview affiliates	42 (13.6)	8 (5.4)	34 (21.1)	<.01
Fairview (HealthEast)	60 (19.4)	32 (21.6)	28 (17.4)	.35
UMN physicians	57 (18.5)	30 (20.3)	27 (16.8)	.43

Abbreviations: NPI, national provider identifier; PDMP, prescription drug monitoring program; UMN, University of Minnesota.

**Figure 2.**
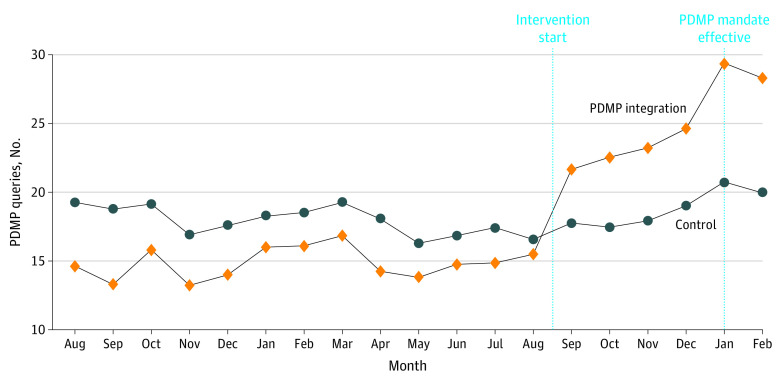
Clinician-Level Monthly Queries to the Prescription Drug Monitoring Program (PDMP), by Study Group

**Figure 3.**
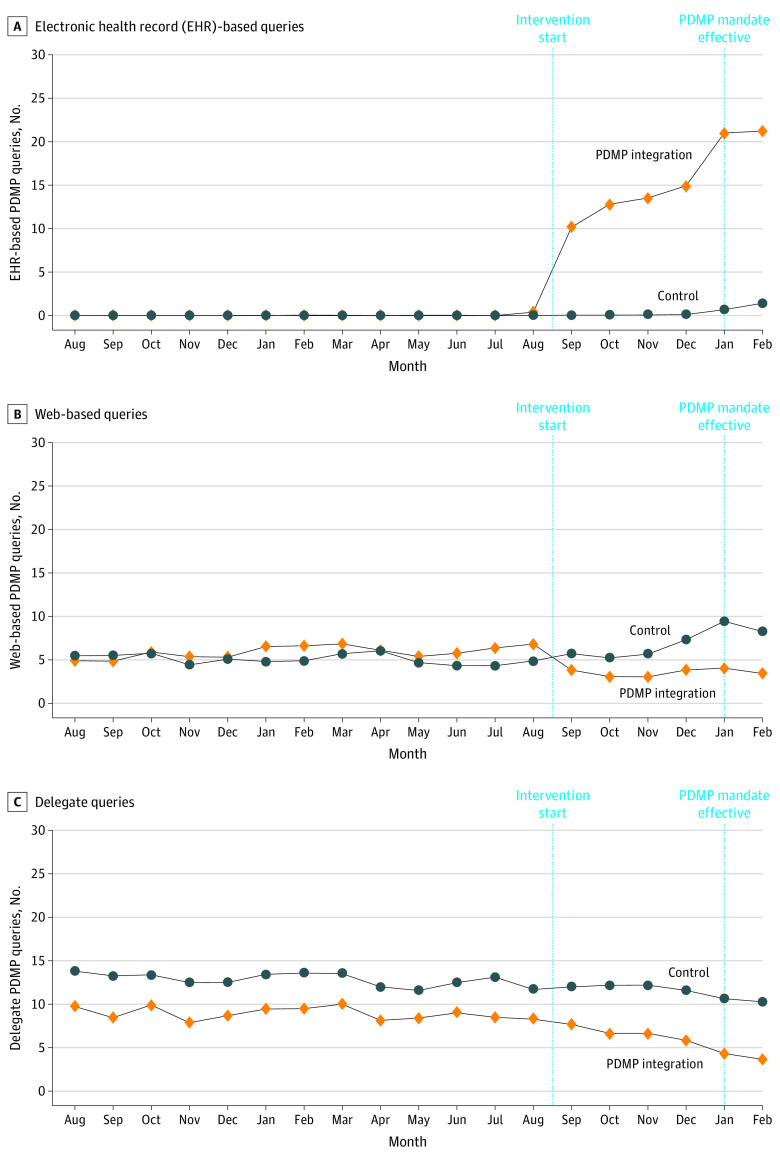
Clinician-Level Monthly Queries to the Prescription Drug Monitoring Program (PDMP), by Study Group and Mode of Access

Figure 2 displays monthly PDMP query counts for clinicians at PDMP integration vs control clinics. Rates of PDMP querying evolved similarly in both clinic groups during the baseline period, diverging only after the intervention. Figure 3 shows that an increase in EHR-integrated PDMP queries drove the increase in total query count among clinicians in intervention clinics, while rates of web-based and delegated queries declined compared with the control group.

Rates of PDMP querying in the baseline and intervention periods by study group are listed in Table 2. Baseline unadjusted monthly average PDMP query counts for the average clinician were 6.6 (95% CI, 4.4-9.9) queries at the control clinics and 8.8 (95% CI, 6.0-13.1) queries for clinicians who received the EHR-integrated PDMP tool at the intervention clinics. During the intervention, PDMP query rates for the typical PCP were 6.9 (95% CI, 4.7-10.3) vs 14.8 (95% CI, 10.0-22.0) queries for clinicians in the control vs PDMP integration clinics, respectively. This represents a relative increase in monthly PDMP query counts of 5% (95% CI, 0%-10%) for clinicians in control clinics and 68% (95% CI, 61%-74%) for clinicians in intervention clinics. Compared with clinics in the control group, the EHR-integrated PDMP tool resulted in a 60% greater increase in the relative change in monthly PDMP queries (95% CI, 51%-70%). Adjusted estimates confirm that an increase in EHR-integrated queries drove the increase in total query count among clinicians in intervention clinics, whereas the EHR-integrated PDMP tool resulted in a 39% greater decrease in the relative change in monthly PDMP queries via the web-based tool or delegates (eTable 1 in Supplement 2). Stratifying by clinician type revealed a similar effect of the intervention on the PDMP querying behavior of physicians compared with nurse practitioners and physician assistants (eTable 2 in Supplement 2). However, the intervention had a larger effect on the PDMP querying behavior of newer clinicians (ie, clinicians with a below-median tenure in practice), compared with clinicians with more experience (eTable 3 in Supplement 2). A model specifying the intervention as a 4-level variable revealed similar, but slightly larger effects in the PDMP-integration-only vs the PDMP-integration-and-choice-architecture groups (eFigure and eTable 4 in Supplement 2).

**Table 2.** Changes in Adjusted PDMP Query Frequency, by Study Group

Query frequency	PDMP integration group, No. (95% CI)		Control group, No. (95% CI)	
	Baseline period	Intervention period	Baseline period	Intervention period
Monthly mean queries per clinician^a^	8.8 (6.0-13.1)	14.8 (10.0-22.0)	6.6 (4.4-9.9)	6.9 (4.7-10.3)
Ratio of mean queries, baseline to intervention^b^	1.68 (1.61-1.74)		1.05 (1.00-1.10)	
Ratio of intervention to control of the change from baseline^b^	1.60 (1.51-1.70)			

Abbreviation: PDMP, prescription drug monitoring program.

^a^ Poisson mixed-effect regression models with clinician and clinic random effects set to zero.

^b^ Poisson mixed-effect regression models described in the Methods section.

## Discussion

Despite the potential benefits for patient care and outcomes, guideline-concordant practices can often be difficult to implement in practice, demanding additional time and effort from clinicians who already face time pressure from full and often chaotic schedules.^16,17,18,19,20^ In this research, we randomized access to an EHR-based intervention designed to reduce the hassle cost of querying the Minnesota PDMP—an essential element of guideline-concordant opioid prescribing. This intervention, which directly integrated access to the Minnesota PDMP into the EHR, potentially alleviated the hassle of querying the PDMP in 2 distinct ways: first by reducing the time cost of querying the PDMP by removing the need to log into a separate PDMP web portal for every query; and second, by lowering the attention cost of remembering to query the PDMP, given that clinicians were prompted when the PDMP data were particularly relevant to their prescribing decision. Reduction in time costs associated with PDMP queries may also have been felt by clinical support staff, given that delegate queries decreased.

Compared with the control group, we found a large and statistically significant increase in PDMP-querying behavior by clinicians who received access to the EHR-integrated PDMP tool. Consistent with the proposition that the EHR-integrated PDMP tool reduced the hassle cost of querying the PDMP, we observed clinicians transition away from previous methods of PDMP querying (ie, web-based and delegated queries) toward the EHR-integrated tool. The effect of the intervention was also larger for less experienced clinicians. This may reflect a less inflexible practice style and/or faster uptake of new features in the EHR; however, the opposite finding could also have been expected if more senior clinicians feel a greater time cost of logging into the web-based PDMP portal for each query.

Of particular interest, the study’s intervention period included the rollout of a statewide mandate requiring clinicians to query the PDMP before prescribing opioids, allowing us to compare the effect of our intervention vs the effect of a legislative mandate. The mandate affected all clinicians at all clinics in our sample, rather than just treatment clinics, and went into effect in January 2021—approximately 4 months into the intervention period. We observed an uptick in PDMP query counts at both intervention and control clinics in January 2021. However, this increase in PDMP query count appeared larger for clinicians at intervention clinics, suggesting a possible reinforcing interaction between our intervention and the mandate. Notably, the uptick following the adoption of the PDMP mandate appeared to be considerably smaller than the changes resulting from the EHR-integrated PDMP tool.

These study findings contribute to a growing body of literature on the effect of behavioral nudges designed to encourage high value care by clinicians.^21^ A subset of behavioral nudges described in the literature aim to change care processes by reducing the time, effort, or attention cost of engaging in high-value practices. These include an intervention that increased generic prescribing by making the generic (rather than the branded drug), the default option in an EHR e-prescribing system.^22^ Relatedly, an intervention that prompted clinicians to actively choose to accept or cancel an order for the influenza vaccine increased influenza vaccination rates significantly.^23^ However, few studies in this literature provide strong evidence via a randomized clinical trial such as the present study.

These study findings also contribute to an active debate on the mechanism by which PDMP-use requirements decreased opioid prescribing. It is possible that PDMP-use mandates increased the information available to clinicians, which may have led to fewer prescriptions. However, PDMP-use mandates may also simply increase the hassle cost of prescribing opioids and other controlled substances. The present study did not directly assess opioid prescribing, but the finding of a larger increase in PDMP queries among clinicians with access to the EHR-integrated tool after Minnesota’s mandated query law was enacted is consistent with what to our knowledge is the only other study available on this topic,^13^ and supports the idea that mandates increase the hassle cost of opioid prescribing.

### Limitations

This study had several limitations, including limited geographic range because we studied a single health care delivery system and its affiliates. Nevertheless, the system and its affiliates included urban, suburban, and rural areas, and as such, the results are likely to be generalizable. Second, consent to access data on clinicians’ PDMP usage was obtained after the start of the intervention. This was because of faulty information provided to the research team regarding data access protocols. However, concerns about potential bias were allayed by a strong overall response rate and no statistically significant difference in response rate across study arms. Third, in our PDMP querying outcome measures, we were unable to distinguish between clinically appropriate and inappropriate queries. Additionally, we lacked data from the Minnesota Board of Pharmacy on the number of opioid prescriptions, so we could not quantify changes in opioid prescribing as a function of access to the EHR-integrated PDMP tool. However, we plan to address this limitation in subsequent analyses of the PRINCE trial interventions, by linking e-prescribing information derived directly from the EHR itself. Finally, within our main effect, we were unable to disentangle the relative importance of the PDMP integration into the EHR vs the reminder function embedded in the Appriss PDMP tool; and therefore, we cannot comment on the relative importance of increasing the salience of the PDMP vs lowering the barriers to accessing it.

## Conclusions

This cluster randomized clinical trial found that direct integration of the PDMP into the EHR increased clinician adherence to recommended prescribing practices for controlled substances such as opioids. Future research should examine the cost and effectiveness of this intervention for promoting safer guideline-concordant opioid prescribing.

## Notes

Supplement 1.
Trial Protocol

Supplement 2. **eTable 1. **Changes in Adjusted Count of PDMP Queries, by Query Modality **eTable 2.** Changes in Adjusted Count of PDMP Queries, by Clinician Type **eTable 3.** Changes in Adjusted Count of PDMP Queries, by Years in Practice **eTable 4.** Changes in Adjusted PDMP Query Frequency, Across 4 Treatment Arms **eFigure. **Clinician-Level Count of PDMP Queries/Month, Across 4 Treatment Arms

Supplement 3.
Data Sharing Statement
