# Supplementary material for: Effect of Integrating Access to a Prescription Drug Monitoring Program Within the Electronic Health Record on the Frequency of Queries by Primary Care Clinicians: A Cluster Randomized Clinical Trial
Source: JAMA Health Forum. 2022 Jun 5;3(6):e221852. doi: 10.1001/jamahealthforum.2022.1852 (PMC9168784; doi:10.1001/jamahealthforum.2022.1852)
Supplement: Supplement 1. — Trial Protocol [file jamahealthforum-e221852-s001.pdf]

PRescribing Interventions for Chronic pain using the Electronic health record (PRINCE): Study Protocol

David M. Vock[a]

Hannah T. Neprash[b]

Alexandra Hanson[c]

Brent A. Elert[c]

David Satin[d]

Alexander J. Rothman[e]

Sonja Short[c]

Pinar Karaca-Mandic[f]

Rebecca Markowitz[g]

Genevieve Melton-Meaux[c,h]

Ezra Golberstein[b] (Corresponding Author, egolber@umn.edu)

[a] Division of Biostatistics, University of Minnesota School of Public Health, Minneapolis, MN, USA.

[b] Division of Health Policy and Management, University of Minnesota School of Public Health, Minneapolis, MN, USA.

[c] Fairview Health Services, Minneapolis, MN, USA.

[d] Department of Family Medicine and Community Health, University of Minnesota Medical School, Minneapolis, MN, USA.

[e] Department of Psychology, University of Minnesota, Minneapolis, MN, USA.

[f] Carlson School of Management, University of Minnesota, Minneapolis, MN, USA

[g] Division of General Internal Medicine, University of Minnesota Medical School, Minneapolis, MN, USA.

[h] Department of Surgery, University of Minnesota Medical School, Minneapolis, MN, USA

Abstract:

Background: Primary care is a frequent source of pain treatment and opioid prescribing. The objective of the Prescribing Interventions for Chronic Pain using the Electronic health record (PRINCE) study is to assess the effects of two behavioral economics-informed interventions embedded within the electronic health record (EHR) on guideline-concordant pain treatment and opioid prescribing decisions in primary care settings.

Methods: The setting for this study is 43 primary care clinics in Minnesota. The study uses a cluster-randomized 2X2 factorial design to test the effects of two interventions. One intervention alters the "choice architecture" within the EHR to nudge clinicians toward non-opioid pain treatment options for opioid-naïve patients and toward tapering when appropriate for patients currently receiving a "high risk" opioid. The other intervention aims to simplify and facilitate checking the prescription drug monitoring program (PDMP) by integrating PDMP access directly within the EHR. Primary outcome for opioid-naïve patients is whether an opioid is prescribed in a primary care visit without currently receiving a non-opioid alternative pain treatment, and primary outcome for current opioid-using patients is whether opioid prescriptions were tapered and whether there was documented rationale for an appropriate taper. An adaptive design allows for the possibility of secondary randomization to test if interventions can be titrated while maintaining efficacy.

Discussion: The PRINCE study will provide real-world evidence on two approaches to improving pain treatment in primary care using the EHR. The adaptive study design strikes a balance between establishing intervention efficacy and testing whether efficacy varies with intervention intensity.

Keywords: Pain, primary care, behavioral economics, pragmatic randomized trials, opioids

Introduction

Chronic pain is highly prevalent, affecting an estimated 11% of the U.S. adult population.[1,2] Opioids are commonly used for treating pain and prescriptions nearly doubled between 2000 and 2010 among all pain visits,[3] with well-known morbidity and mortality consequences.[4] In March 2016, the U.S. Centers for Disease Control and Prevention (CDC) published guidelines for treating chronic pain in adults who are outside of cancer, palliative, and end-of-life care settings.[5,6] The goal of the guidelines is to improve patient-clinician communications around the safety and effectiveness of pain treatment, including reducing opioid use where clinically appropriate. Primary care is a key target for improving treatment of chronic pain and reducing risks from inappropriate opioid use. Non-malignant pain is among the most common reasons for primary care visits,[7] and primary care practitioners (PCPs) account for nearly half of all opioid prescriptions.[8] Primary care prescribing frequently does not adhere to clinical guidelines.[9-11] As such, it is important to identify effective ways of changing treatment and prescribing behavior of PCPs to be more concordant with clinical guidelines.

A recent literature studies how Electronic Health Records (EHR)-based “nudges” affect clinician opioid prescribing and pain treatment decisions. “Nudges” rely on behavioral science insights to encourage certain behaviors without direct compulsion or financial incentives. For example, two recent studies found that reducing the EHR’s default number of pills per opioid prescription order led to fewer opioids prescribed after surgical procedures and at emergency department discharge, whereas other research found that safety alerts in the EHR for when a patient was co-prescribed an opioid and a benzodiazepine was not associated with changes in prescribing patterns.[12-14]

The CDC's chronic pain guidelines call for prescribers to check their state-administered prescription drug monitoring program (PDMP) to identify potentially problematic opioid use patterns. Some studies examine the effects of mandating clinicians to check the PDMP.[15,16] Other research finds that many clinicians do not check the PDMP when prescribing opioids, or do so relatively infrequently.[17-19] PDMP queries generally require clinician time and effort to use a website outside of the EHR and then search for relevant information, and these hassles may contribute to underuse of PDMPs.[18,19] Limited evidence exists on the effects of PDMP integration into the EHR.[20] We are unaware of any randomized trials of PDMP integration into the EHR.

In the PRescribing Interventions for Chronic pain using the Electronic health record (PRINCE) study we use a cluster-randomized 2X2 factorial design to test the effects of two interventions designed to improve pain treatment in primary care, consistent with CDC guidelines, using EHR-based interventions that rely on behavioral economics principles.

Methods

Conceptual Framework

Clinicians make decisions within structures that can either facilitate or inhibit best practices, and behavioral economics-informed interventions aim to improve these structures. Clinical decisions occur under situational constraints that may limit deliberation,[21] which may increase reliance on cognitively-accessible decision rules or heuristics. If PCPs develop prescription routines or defaults that favor opioids, PCPs may benefit from modest changes in the structure of their decision process that prompt deliberation and highlight the broader set of treatment options for

pain. Similar intervention strategies have been shown to disrupt suboptimal routines in other domains.[22,23] Specifically, EHR-based prompts or reminders that make salient the CDC recommendations regarding opioid prescribing could increase PCPs' consideration of non-opioid treatments and when combined with the provision of an easy-to-use, pre-populated set of non-opioid options may increase the rate at which they are ordered. Moreover, presenting that information within the EHR at the time of prescribing medications places limited additional disruptions to the clinician's workflow.

Intervention strategies can also make it less difficult for clinicians to access or utilize information for appropriate pain treatment decisions. Unnecessary points of friction can undermine the likelihood of pursuing or completing a task, even one that is construed favorably and people indicate they will complete.[24] For instance, clinicians may affirm the value of information about a patient's prescribing history, yet not use that information because it is difficult to access. Thus, strategies that remove impediments to this information may facilitate clinicians delivering care that is guideline-supported and concordant with their intentions.

Study Setting

The setting for this study is 43 primary care clinics that are owned by Fairview Health Services, University of Minnesota Physicians, or are independent affiliate clinics of Fairview Health Services. 39 of these clinics are in the Minneapolis-St. Paul metropolitan area and four clinics are in Greater Minnesota. All clinics use an Epic EHR system.

Description of interventions

The PRINCE study investigates two interventions that were informed by behavioral economics principles. These interventions were chosen based on input from PCPs, pain treatment, and opioid use stakeholders in the Fairview system.

The first intervention (the "choice architecture" intervention) is two alerts within Epic that the study team designed based on choice architecture principles from behavioral economics. One alert is triggered for opioid-naïve patients (defined as patients without an active opioid prescription in the past 6 months) when a PCP initiates the process of signing an opioid prescription order. This alert (Figure 1) reminds PCPs that opioids should only be prescribed when necessary, in the lowest effect dose, and for the shortest duration necessary after non-opioid options have been considered. The alert also offers a pre-populated "SmartSet" with options to order both pharmacological and non-pharmacological non-opioid pain treatment alternatives. The other alert applies to visits for patients currently prescribed a "high-risk" opioid (defined as a current maximum morphine milligram equivalent (MME) of 50 or higher, or currently prescribed an opioid and a benzodiazepine) and is triggered when a PCP initiates the process of signing an order for a refill or any other opioid prescription. This alert (Figure 2) reminds that the CDC recommends tapering opioid prescriptions if clinically appropriate, and presents PCPs with a calculation of what a 10% reduction in MME would be, relative to the patient's current prescription. The alerts were pilot-tested for usability by five clinicians who are not included in the subsequent trial, and the final alerts were modified in response to pilot-testers' feedback.

Figure 1. Choice Architecture Intervention for Opioid-Naïve patients

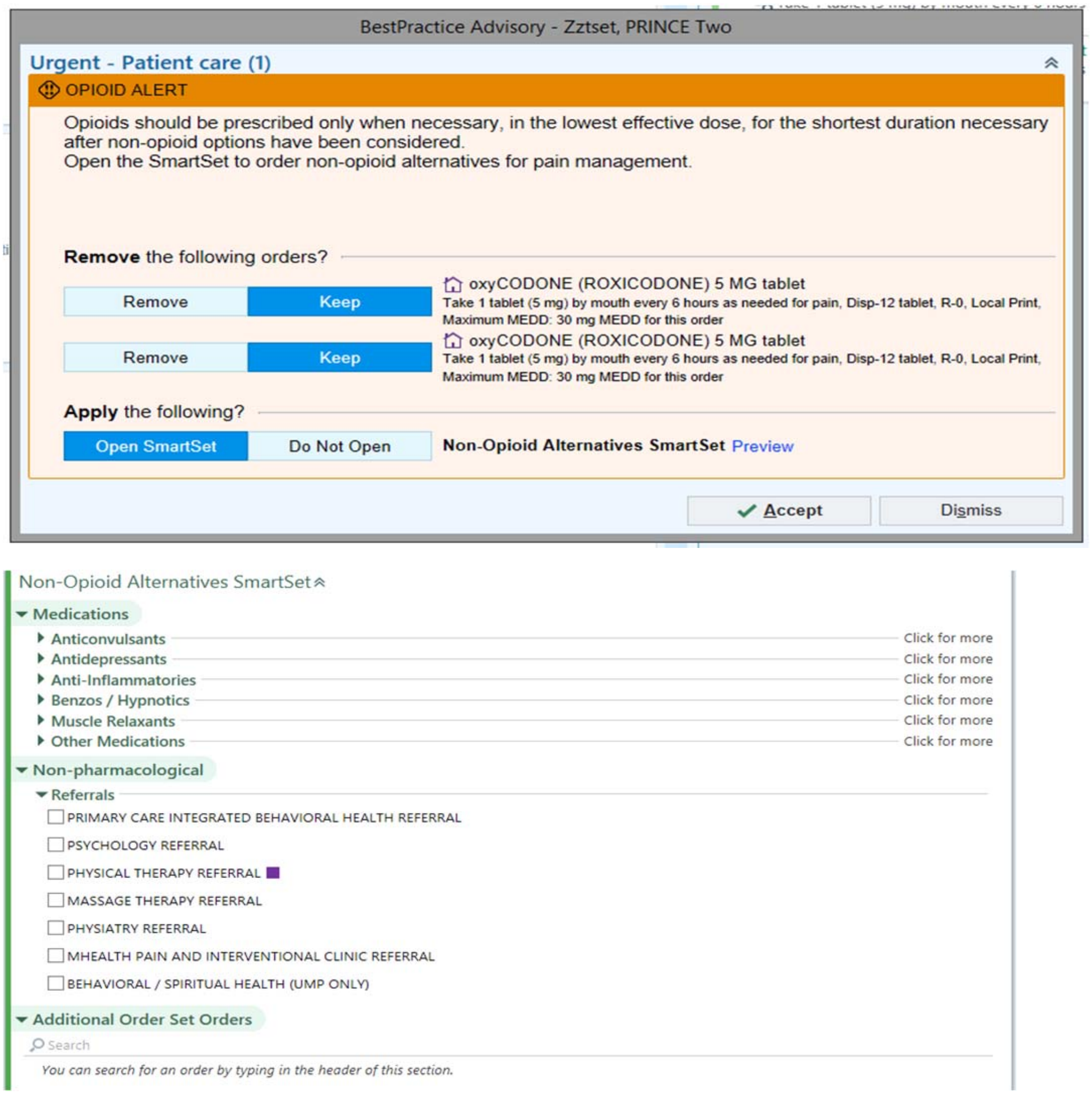

Figure 2. Choice Architecture Intervention for Current "High Risk" Opioid Using Patients

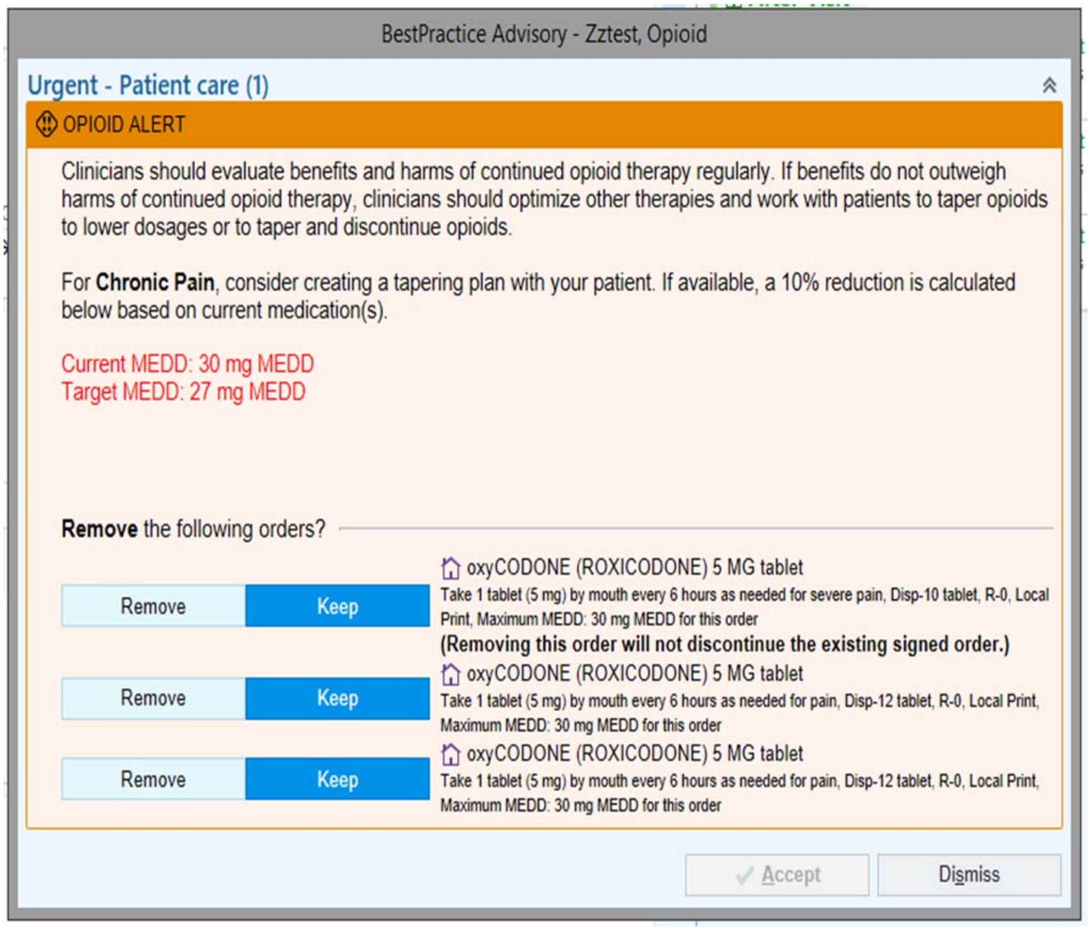

The second intervention integrates direct access to a patient's Minnesota PDMP record into Epic, using the Appriss PMP Gateway tool. This proprietary tool uses a single-sign-on allowing clinicians to query a patient's controlled substance prescription and dispensing history as recorded in the Minnesota PDMP directly from the patient's record in Epic (Figure 3). The tool presents information about the history of narcotics, sedatives, and stimulants prescribed to the patient and by prescriber, along with a calculated "overdose risk score." (Figure 4) An alert reminds a prescriber that the PDMP needs review if a patient is being seen who has at least three opioid prescriptions in the past year and at least one in the past six months.

Figure 3. Integration of PDMP Access within the EHR

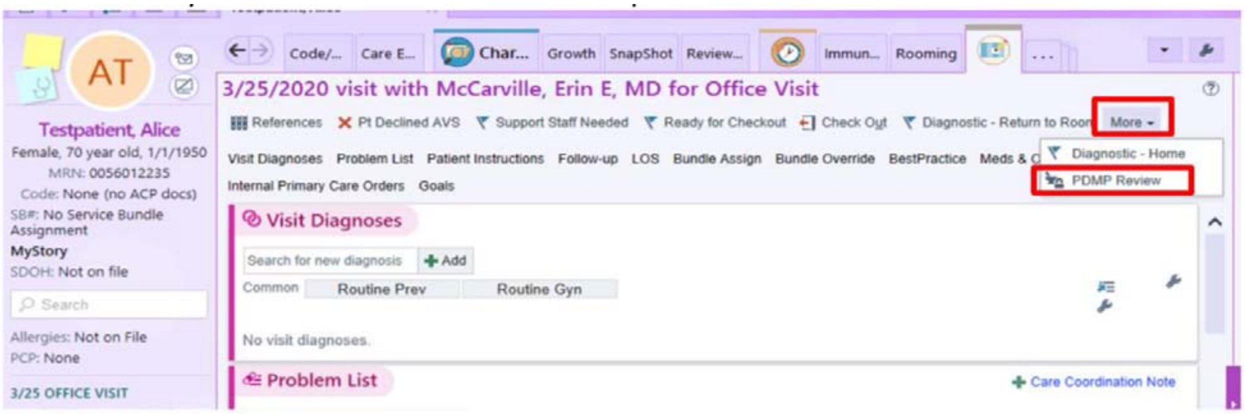

Figure 4. Presentation of PDMP Information in Appriss PMP Gateway Tool

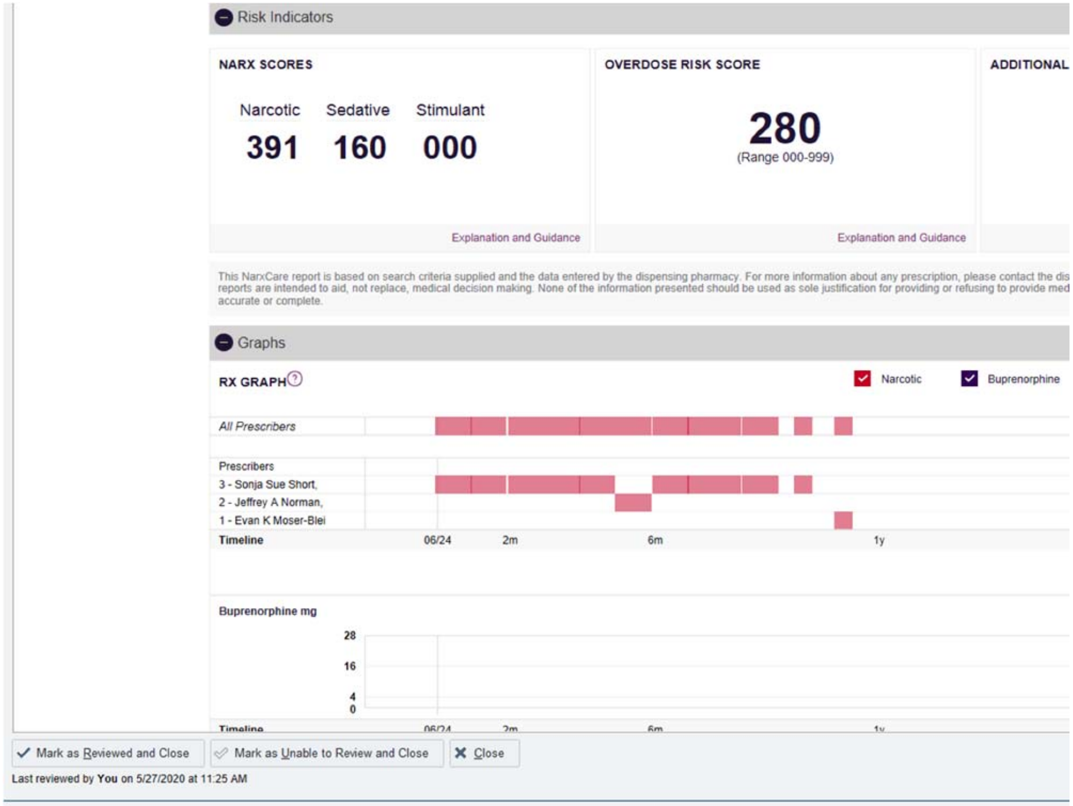

The delivery system will implement the interventions in the same way that EHR enhancements and changes are implemented normally. “Tip sheets” about the interventions and how to use

them, will be shared with all PCPs in the appropriate intervention arm clinics by their clinic leadership (included in the Supplemental Materials).

Overview of study design and randomization

A clinic-randomized factorial design will be used to test the effects of the two interventions on pain treatment and opioid prescribing decisions by PCPs. The design contains up to two stages of randomization (Figure 5). In the first stage, 43 primary care clinics will be randomized 1:1:1:1 to receive the “choice architecture” intervention alone, the PDMP integration intervention, both interventions, or care as usual. All PCPs (physicians, physicians assistants, and nurse practitioners) working in the study clinics were exposed to the intervention(s) that their clinic is assigned to. To ensure similar characteristics of the clinics across randomized groups, covariate constrained randomization procedures will be used.[25,26] Specifically, randomization is constrained so that the distribution of clinic system (Fairview, HealthEast, University of Minnesota Physicians, or Fairview affiliate clinic) was equal across each of the 4 intervention arms and within levels of each intervention marginally and to ensure equal number of clinics within each arm overall.

Figure 5. Study Design

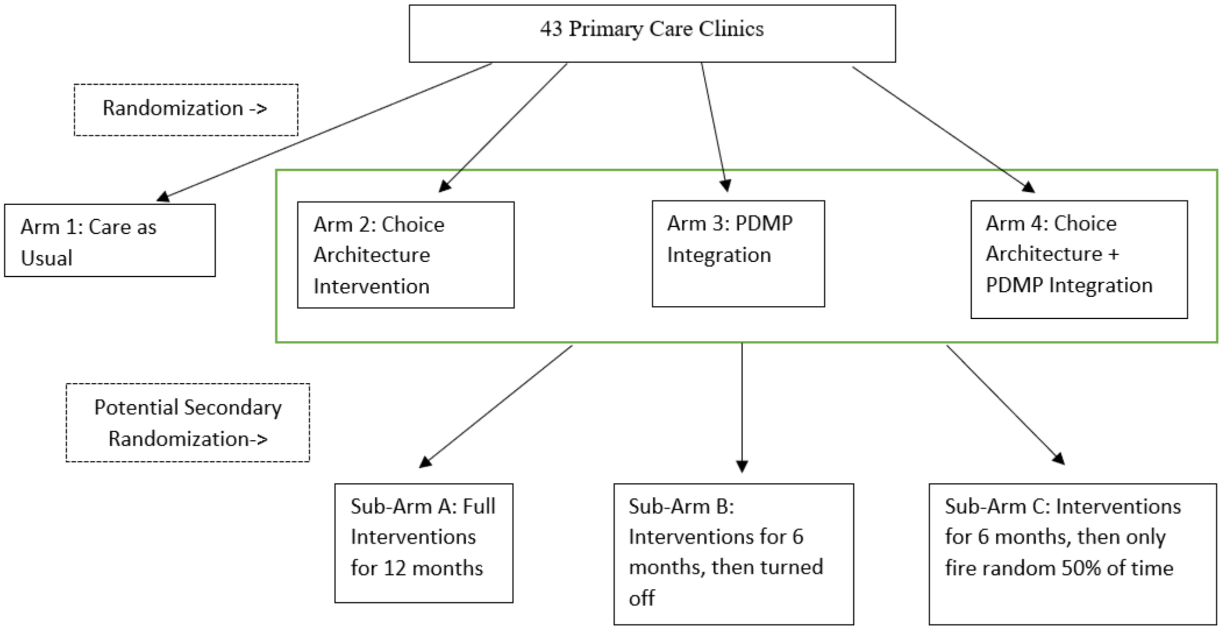

The intervention period will last for 12 months. An interim outcomes analysis is planned to inform a possible second stage of randomization after six months, when the study team will consider re-randomizing the clinics receiving any active intervention to reduced intensity (i.e., turned off after six months, or interventions fire only half the time when triggered) or to remain with the original intensity. This second stage of randomization will allow for testing whether effects of the interventions can be sustained with reduced intensity. The decision to proceed with the second randomization will be made in consultation with the external data monitoring committee (described below).

We did not adopt a fixed design where the second stage of randomization would automatically proceed for several reasons. First, the efficacy of both interventions has not been established previously; our primary goal was to establish whether these interventions have efficacy. We did not want to reduce the likelihood of demonstrating that the interventions are efficacious by re-randomizing a subset of the clinics to interventions at lower intensity. Second, though our power

calculations indicated that we had sufficient power to detect even modest intervention effects with the first 6 months of data, these calculations were sensitive to assumptions about the intra-provider and intra-clinic correlation as well as each PCP's monthly number of encounters. The COVID-19 pandemic reduced confidence in these assumptions. Thus, if either intervention does not show a significant effect during the first approximately half of the study (either because the study was underpowered or the intervention had a smaller effect than anticipated), we will not do a secondary randomization of clinics.

Aims & Hypotheses

The primary aim of the study is to test whether two separate interventions affected PCP decisions around pain treatment and opioid prescription. We hypothesize that each of the interventions will increase the likelihood that pain treatment and opioid prescribing decisions will be consistent with clinical guidelines. Specifically, we hypothesize that each of the interventions will reduce initiation of opioid-prescriptions without a concurrent or previous non-opioid recommended treatment among patients who are opioid naïve. Similarly, we hypothesize that each intervention will lead to increased use of appropriate opioid tapering among patients currently prescribed a "high risk" opioid. We also will examine several secondary outcomes representing more-specific features of opioid prescribing and pain treatment.

Secondary aims include (1) assessing whether the study interventions affected the frequency with which PCPs checked the Minnesota PDMP (2) assessing the effect of the interventions on PCP attitudes and perceptions of the quality of clinical decision support for pain treatment and opioid prescribing in the EHR, satisfaction with the PDMP, and whether frequency of engaging patients in discussions about opioids changed in 12 months and (3) assessing whether exposure to the

interventions firing less-frequently affects outcomes differently than when the intervention fires whenever triggered.

As an exploratory aim, we will assess potential moderators of the intervention as well as assess whether there is an interaction between the two interventions being studied. We hypothesize that the two interventions will interact to have a stronger effect on the study outcomes.

Study Participants, Recruitment, and Consent

The University of Minnesota IRB approved this study with a waiver of informed consent of both PCPs and patients for being exposed to the study interventions and for the study team to retrospectively analyze EHR data for all patient encounters with a PCP practicing in the study clinics. PCPs are not blinded to the interventions. Four clinics in Fairview Health Services were excluded from the study because they had already adopted PDMP integration, but all other primary care clinics in the systems are included.

Electronic informed consent was obtained from PCPs for participating in pre- and 12-month post-intervention web surveys and to authorize study investigators to receive data about frequency of checking the Minnesota PDMP from the Minnesota Board of Pharmacy. All PCPs in the 43 study clinics are eligible to participate in the web surveys and PDMP data authorization, except for resident physicians who are not allowed to receive compensation for completing the web survey and first-year residents who do not have prescriptive authority, respectively.

Cohorts, Measures, and Outcomes

Because the choice architecture arm of the study uses separate alerts for opioid-naïve and current, "high-risk" opioid-using patients, there are separate primary outcomes for visits with each of those populations. The data for those outcomes are at the encounter-level and for all cohorts includes patient visits during the 12 months before and after the start of the study interventions for patients 18 and older who did not have a current cancer diagnosis.

For the opioid-naïve population, the analysis cohort includes patient visits meeting the above criteria who did not have an active opioid prescription within the previous six months from the date of the visit. To focus on visits where opioids are potentially a treatment option, an additional inclusion criterion for the analytic sample is having a visit diagnosis that was associated with at least a 3% chance of an opioid prescription among opioid-naïve patients, and there were at least 25 opioid orders in visits with the diagnosis for all visits in the study clinics during the 12-month period before the study, excluding diagnoses for current opioid use. This list of 294 diagnoses includes many chronic pain-related diagnoses and is provided in the supplemental materials. The primary outcome for the opioid-naïve population is a binary variable for whether an opioid is prescribed in a primary care visit, without currently receiving a non-opioid alternative pain treatment (including a new order for a non-opioid pain treatment). Three secondary outcomes for this population include 1) Whether a CDC-recommended non-opioid treatment was ordered in a primary care visit (e.g., referral to physical therapy, prescription of an NSAID, referral to pain clinic). 2) The length of an opioid prescription. 3) The MME of an opioid prescription. These outcomes are all derived entirely from EHR data.

For the current "high risk" opioid population, the analysis cohort included primary care visits for patients with a current opioid prescription with an MME of 50 or higher, or currently prescribed an opioid and a benzodiazepine. The primary outcome for this population is a variable with three

mutually-exclusive categories. Category 1 (appropriate taper): Whether a patient visit had an order that would reduce MME by no greater than 20%, relative to the current prescription, and there is documented evidence that the reduction is consistent with CDC guidelines. Category 2 ("inappropriate" taper): Whether a patient visit had an order that would reduce MME without documented evidence that the reduction was consistent with CDC guidelines, or, decreased MME by greater amounts than recommended (>20% relative reduction in MME). Category 3 (no taper): Whether patient visit had no reduction in MME. Data for this outcome are derived from EHR data. For encounters in which the MME was reduced by no more than 20%, members of the study team blinded to randomization will audit the chart to assess whether the taper was consistent with CDC guidelines (the chart audit tool is included in the supplemental materials). Two secondary outcomes specific to this population include 1) Whether there is a partial reduction in the MME or prescription length of refill orders, versus a total opioid discontinuation. 2) Whether there is an increase in the MME/day.

Another secondary study outcome is the number of times each PCP checked the Minnesota PDMP per calendar month. The data come from the Minnesota Board of Pharmacy that administers the state's PDMP and are available for all PCPs who authorized access to this information. The total number of PDMP checks per month include checks that the PCP does through the PDMP web portal, checks through the web portal that were done by a delegate on the PCP's behalf, and checks that were done using the integrated PDMP tool within the EHR. Data will be available for the 12 months prior to the study and the 12-month study period.

Seven measures from web surveys of PCPs are secondary outcomes. These outcomes relate to PCP satisfaction with alerts and clinical decision support for pain treatment and opioid

prescribing, and around satisfaction with using the PDMP. (Full web survey instruments are in the supplemental material.)

The research team will collect several other measures to be used as covariates which are described below.

Data collection and Security

Access to the EHR data for the research team is through the secure data shelter of the University of Minnesota's Biomedical Informatics and Data Access Core. Web survey data are collected via the RedCAP system and stored in a secure server. Data on Minnesota PDMP usage will be securely transferred from the Minnesota Board of Pharmacy and stored on a secure server.

Primary Outcome Analysis

To test our primary hypotheses, we will fit mixed effects logistic regression models using data from the 12-month pre-intervention period, and the post-intervention period prior to any second-stage randomization (the model for the current "high risk" opioid group will be multinomial logistic regression due to the 3-category outcome). All models will include fixed effects for whether the encounter was at an intervention arm clinic, an indicator for whether the measurement was after the interventions began, and their interaction. The interaction is the primary measure of the intervention effect. These models include random effects for clinic and PCP (nested in clinic) to account for within-clinic and within-provider correlation. Because of the cluster-randomized design with a moderate number of clinics, residual imbalances in patient and PCP-characteristics among the randomized groups may exist. The primary analysis model will adjust for characteristics at all levels of the hierarchy including clinic-level (indicators for the four systems), PCP-level (clinician type [MD/DO, NP, or PA], sex, and length of tenure in

medicine), and patient-level (age, sex, race/ethnicity, insurance status, and in-person or virtual visit). Separate models will be fit to assess the PDMP integration and choice architecture intervention and for the opioid-naïve and current, high-risk opioid users. In each model, the intervention group pools across two arms of the factorial design (e.g., the choice architecture intervention pools across arms 2 and 4) and the control condition pools across the remaining two groups (e.g., arms 1 and 3 to continue the example). A Bonferroni correction will adjust inference for multiple comparison across two different populations (opioid naïve and current, high-risk opioid users) but we will not adjust for multiple tests across the two different interventions.

As an exploratory analysis, we will assess whether PCP characteristics (clinician type and tenure in medicine) and patient characteristics (age, sex, and race/ethnicity) moderate the interventions' effect. Tests for treatment effect heterogeneity will be implemented by adding the interaction between the potential moderator and the intervention indicator to the models for the primary outcome (adjusting for the same factors described above). The effect of the intervention within subgroups formed from the potential moderators will be estimated by fitting separate models within each subgroup.

Subgroup analyses will not be adjusted for multiple comparisons; they are supportive to the primary outcome analysis. Subgroup analyses will be interpreted with caution due to limited power and uncontrolled type I error.

If the study proceeds to the second stage of randomization, we will fit mixed effects logistic regression models using data from 12 months pre- and post-intervention. All models will include fixed effects for intervention arm (control, continued intervention, 50% intervention, or intervention turned-off), indicators for whether the measurement was after the initial

interventions began or after the second stage randomization, and their interaction. The interaction is the primary measure of the intervention effect. The models will include the same random effects and covariate adjustment as above.

Secondary Outcomes Analysis: PDMP and Web Survey data

For other EHR-derived secondary outcomes, we will use the same general modeling framework as for the primary outcome but will fit mixed effects logistic or linear models depending on whether the outcome was categorical or continuous.

To assess intervention effects on the frequency of checking the PDMP we will fit mixed effects Poisson regression models using data from the 12 months pre- and post-intervention. All models will include fixed effects for whether the PCP was at an intervention arm clinic, indicator for whether the month was during the intervention period, and their interaction. The models will adjust for clinician type, length of tenure in medicine, and health system indicators, and include random effects for clinic and PCP (nested in clinic) to account for within-clinic and within-provider correlation. As an exploratory analysis, we will assess whether PCP characteristics (clinician type and length of tenure) moderate the effect of the interventions using a similar process as the primary outcome.

A similar approach will be used for the web survey data, except we will fit mixed effect linear regression models using data from the pre- and post-intervention surveys. All models will include fixed effects for whether the PCP was at an intervention arm clinic, an indicator for whether the survey was the 12-month follow-up, and their interaction.

Interim monitoring

The University of Minnesota IRB and the study sponsor agreed that a Data Safety and Monitoring Board was unnecessary. However, a Data Monitoring Committee (DMC) will be formed and asked to recommend graduation to the second stage of randomization if there is clear and substantial evidence of treatment efficacy. As a guideline, the Lan-DeMets spending function analog of the Pocock boundaries will be used to monitor the primary outcome comparison for the primary aims.[27] The study team, with the exception of the unblinded statistician, will be blinded to interim outcomes. We plan to conduct a single interim analysis occurring midway through the study.

The interim analysis of the effect of the interventions on key secondary outcomes will be provided to the DMC. Should the stopping boundary be crossed with clear evidence of an intervention's effect, we will ask the DMC to recommend proceeding to the secondary randomization of intervention arm. Without clear evidence of an intervention's effect, we will continue the study for an additional 6 months without re-randomizing any clinics (to ensure sufficient power to detect an effect of the intervention). The decision to move to the second stage of randomization will be made separately for both intervention arms. If the PDMP integration intervention shows substantial efficacy at the interim analysis, we will recommend that the PDMP integration be made available to all clinics. A decision on what constitutes substantial efficacy will be made in consultation with the DMC. As the study interventions have small potential adverse effects, the DMC would not be asked to recommend early termination for futility.

Summary

The PRINCE study will use a clinic-randomized factorial design with potential for secondary randomization to test the effects of two separate behavioral economics-informed interventions on

a range of outcomes related to guideline-concordant practices of opioid prescribing and pain treatment. One limitation of the study is that the primary outcome for the current-opioid using population relies on audits of medical charts to classify opioid tapers as appropriate or inappropriate. This classification may miscode some tapers as inappropriate to the extent that the clinical rationale was not entered into the patient's chart.

The study contains several novel aspects. One intervention arm includes two novel interventions to the choice architecture around opioid prescribing, and another intervention arm represents an already-diffusing EHR tool to facilitate PDMP use that has not yet been rigorously evaluated. An adaptive design will test whether the interventions can be "titrated" without compromising efficacy. We considered a fixed design where all active intervention clinics would be re-randomized to the different intensities of the intervention. Such a design would enable simultaneous testing of (a) whether the interventions are efficacious compared to control and (b) whether effects of the interventions can be sustained with reduced (or no) intensity after an initial 6-month roll-out. That experimental framework would provide useful information for broad-scale implementation and designing future EHR nudges. However, that re-randomization to a reduced-intensity intervention reduces power for the primary comparison of the interventions (at full intensity) to control. Well-designed adaptive trials in which clinics are re-randomized only if the interventions demonstrate significant efficacy provide a compelling framework to balance these two competing interests and should be considered for future studies.

Acknowledgements

Funding: This work was supported by the National Institute of Health [grant numbers 5R21DA046084, 5R33DA046084].

References

1. Nahin RL. Estimates of pain prevalence and severity in adults: United States, 2012. *J Pain.* 2015;16(8):769-780.
2. Johannes CB, Le TK, Zhou X, Johnston JA, Dworkin RH. The prevalence of chronic pain in United States adults: results of an Internet-based survey. *J Pain.* 2010;11(11):1230-1239.
3. Schieber LZ, Guy GP, Jr., Seth P, et al. Trends and Patterns of Geographic Variation in Opioid Prescribing Practices by State, United States, 2006-2017. *JAMA Netw Open.* 2019;2(3):e190665.
4. Rudd RA, Aleshire N, Zibbell JE, Gladden RM. Increases in Drug and Opioid Overdose Deaths--United States, 2000-2014. *MMWR Morb Mortal Wkly Rep.* 2016;64(50-51):1378-1382.
5. Dowell D, Haegerich TM, Chou R. CDC Guideline for Prescribing Opioids for Chronic Pain--United States, 2016. *JAMA.* 2016;315(15):1624-1645.
6. Dowell D, Haegerich TM, Chou R. CDC Guideline for Prescribing Opioids for Chronic Pain - United States, 2016. *MMWR Recomm Rep.* 2016;65(1):1-49.
7. Daubresse M, Chang HY, Yu Y, et al. Ambulatory diagnosis and treatment of nonmalignant pain in the United States, 2000-2010. *Med Care.* 2013;51(10):870-878.
8. Levy B, Paulozzi L, Mack KA, Jones CM. Trends in Opioid Analgesic-Prescribing Rates by Specialty, U.S., 2007-2012. *Am J Prev Med.* 2015;49(3):409-413.
9. Morasco BJ, Duckart JP, Dobscha SK. Adherence to clinical guidelines for opioid therapy for chronic pain in patients with substance use disorder. *J Gen Intern Med.* 2011;26(9):965-971.
10. Neprash HT, Gaye M, Barnett ML. Abrupt Discontinuation of Long-term Opioid Therapy Among Medicare Beneficiaries, 2012-2017. *J Gen Intern Med.* 2021;36(6):1576-1583.
11. Tournebize J, Gibaja V, Muszczak A, Kahn JP. Are Physicians Safely Prescribing Opioids for Chronic Noncancer Pain? A Systematic Review of Current Evidence. *Pain Pract.* 2016;16(3):370-383.
12. Chiu AS, Jean RA, Hoag JR, Freedman-Weiss M, Healy JM, Pei KY. Association of Lowering Default Pill Counts in Electronic Medical Record Systems With Postoperative Opioid Prescribing. *JAMA Surg.* 2018;153(11):1012-1019.
13. Montoy JCC, Coralic Z, Herring AA, Clattenburg EJ, Raven MC. Association of Default Electronic Medical Record Settings With Health Care Professional Patterns of Opioid Prescribing in Emergency Departments: A Randomized Quality Improvement Study. *JAMA Intern Med.* 2020.
14. Smith LB, Golberstein E, Anderson K, et al. The Association of EHR Drug Safety Alerts and Co-prescribing of Opioids and Benzodiazepines. *J Gen Intern Med.* 2019;34(8):1403-1405.
15. Schuler MS, Heins SE, Smart R, et al. The state of the science in opioid policy research. *Drug Alcohol Depend.* 2020;214:108137.
16. Carey CM, Meille G, Buchmueller TC. Provider Compliance With Kentucky's Prescription Drug Monitoring Program's Mandate To Query Patient Opioid History. *Health Aff (Millwood).* 2021;40(3):461-468.

17. Irvine JM, Hallvik SE, Hildebran C, Marino M, Beran T, Deyo RA. Who uses a prescription drug monitoring program and how? Insights from a statewide survey of Oregon clinicians. *J Pain.* 2014;15(7):747-755.
18. Rutkow L, Turner L, Lucas E, Hwang C, Alexander GC. Most primary care physicians are aware of prescription drug monitoring programs, but many find the data difficult to access. *Health Aff (Millwood).* 2015;34(3):484-492.
19. Blum CJ, Nelson LS, Hoffman RS. A survey of Physicians' Perspectives on the New York State Mandatory Prescription Monitoring Program (ISTOP). *J Subst Abuse Treat.* 2016;70:35-43.
20. Holmgren AJ, Apathy NC. Evaluation of Prescription Drug Monitoring Program Integration With Hospital Electronic Health Records by US County-Level Opioid Prescribing Rates. *JAMA Netw Open.* 2020;3(6):e209085.
21. Tsiga E, Panagopoulou E, Sevdalis N, Montgomery A, Benos A. The influence of time pressure on adherence to guidelines in primary care: an experimental study. *BMJ Open.* 2013;3(4).
22. Rothman AJ, Gollwitzer PM, Grant AM, Neal DT, Sheeran P, Wood W. Hale and Hearty Policies: How Psychological Science Can Create and Maintain Healthy Habits. *Perspect Psychol Sci.* 2015;10(6):701-705.
23. Wood W, Neal DT. Healthy Through Habit: Interventions for Initiating and Maintaining Health Behavior Change. *Behavioral Science and Policy.* 2016;2(1):71-83.
24. Sunstein CR. Sludge Audits. *Behavioural Public Policy.* 2020(1-20).
25. Carman WF, Elder AG, Wallace LA, et al. Effects of influenza vaccination of health-care workers on mortality of elderly people in long-term care: a randomised controlled trial. *Lancet.* 2000;355(9198):93-97.
26. Dickinson LM, Beaty B, Fox C, et al. Pragmatic Cluster Randomized Trials Using Covariate Constrained Randomization: A Method for Practice-based Research Networks (PBRNs). *J Am Board Fam Med.* 2015;28(5):663-672.
27. DeMets DL, Lan KK. Interim analysis: the alpha spending function approach. *Stat Med.* 1994;13(13-14):1341-1352; discussion 1353-1346.
