# Supplementary material for: Effect of Integrating Access to a Prescription Drug Monitoring Program Within the Electronic Health Record on the Frequency of Queries by Primary Care Clinicians: A Cluster Randomized Clinical Trial
Source: JAMA Health Forum. 2022 Jun 5;3(6):e221852. doi: 10.1001/jamahealthforum.2022.1852 (PMC9168784; doi:10.1001/jamahealthforum.2022.1852)
Supplement: Supplement 2. — eTable 1. Changes in Adjusted Count of PDMP Queries, by Query Modality eTable 2. Changes in Adjusted Count of PDMP Queries, by Clinician Type eTable 3. Changes in Adjusted Count of PDMP Queries, by Years in Practice eTable 4. Changes in Adjusted PDMP Query Frequency, Across 4 Treatment Arms eFigure. Clinician-Level Count of PDMP Queries/Month, Across 4 Treatment Arms [file jamahealthforum-e221852-s002.pdf]

Supplemental Online Content

Neprash HT, Vock DM, Hanson A, et al. Effect of integrating access to a prescription drug monitoring program within the electronic health record on the frequency of queries by primary care clinicians: a cluster randomized clinical trial. *JAMA Health Forum*;3(6):e221852.
doi:10.1001/jamahealthforum.2022.1852

eTable 1. Changes in Adjusted Count of PDMP Queries, by Query Modality

eTable 2. Changes in Adjusted Count of PDMP Queries, by Clinician Type

eTable 3. Changes in Adjusted Count of PDMP Queries, by Years in Practice

eTable 4. Changes in Adjusted PDMP Query Frequency, Across 4 Treatment Arms

eFigure. Clinician-Level Count of PDMP Queries/Month, Across 4 Treatment Arms

This supplemental material has been provided by the authors to give readers additional information about their work.

© 2022 Neprash HT et al. *JAMA Health Forum.*

EHR-Integrated PDMP Tool

While the Prescribing Interventions for Chronic pain using the Electronic health record (PRINCE) study cluster-randomized two interventions designed to improve pain treatment in primary care, this manuscript focuses on the intervention that integrated direct access to a patient's Minnesota PDMP record into Epic, using the Appriss PMP Gateway tool. This proprietary tool uses a single-sign-on allowing clinicians to query a patient's controlled substance prescription and dispensing history as recorded in the Minnesota PDMP directly from the patient's record in Epic. The tool presents information about the history of narcotics, sedatives, and stimulants prescribed to the patient and by prescriber, along with a calculated "overdose risk score." An alert reminds a prescriber that the PDMP needs review if a patient is being seen who has at least three opioid prescriptions in the past year and at least one in the past six months.

© 2022 Neprash HT et al. *JAMA Health Forum.*

eTable 1. Changes in Adjusted Count of PDMP Queries, by Query Modality

Characteristic	PDMP Integration		No PDMP Integration	
	Baseline Period	Intervention Period	Baseline Period	Intervention Period
	Web-Based & Delegate PDMP Queries			
Monthly PDMP queries, mean (95% CI)	7.9 (5.3-11.8)	5.0 (3.3-7.4)	6.4 (4.2-9.8)	6.6 (4.4-9.9)
Ratio of Mean PDMP queries, baseline to intervention (95% CI)	0.63 (0.60-0.67)		1.03 (0.98-1.07)	
Ratio between PDMP integration and control of the change from baseline (95% CI)	0.61 (0.57-0.66)			

© 2022 Neprash HT et al. *JAMA Health Forum.*

eTable 2. Changes in Adjusted Count of PDMP Queries, by Clinician Type

Characteristic	PDMP Integration		No PDMP Integration	
	**Baseline Period**	**Intervention Period**	**Baseline Period**	**Intervention Period**
	Physicians			
Monthly PDMP queries, mean for typical PCP(95% CI)	9.3 (6.0-14.4)	14.4 (9.3-22.4)	7.9 (5.0-12.5)	7.9 (5.1-12.4)
Ratio of Mean PDMP queries, baseline to intervention (95% CI)	1.55 (1.48-1.62)		1.00 (0.96-1.06)	
Ratio between PDMP integration and control of the change from baseline (95% CI)	1.55 (1.45-1.65)			
	Nurse Practitioners and Physician Assistants			
Monthly PDMP queries, mean for typical PCP(95% CI)	8.7 (6.2-12.3)	18.1 (12.8-25.5)	4.8 (3.3-7.2)	6.3 (4.3-9.3)
Ratio of Mean PDMP queries, baseline to intervention (95% CI)	2.07 (1.91-2.24)		1.31 (1.16-1.47)	
Ratio between PDMP integration and control of the change from baseline (95% CI)	1.58 (1.37-1.82)			

© 2022 Neprash HT et al. *JAMA Health Forum.*

eTable 3. Changes in Adjusted Count of PDMP Queries, by Years in Practice

Characteristic	PDMP Integration		No PDMP Integration	
	Baseline Period	Intervention Period	Baseline Period	Intervention Period
	Clinicians with Above-Median Tenure in Medicine (≥13 years)			
Monthly PDMP queries, mean for typical PCP(95% CI)	9.1 (5.6-14.7)	13.0 (8.0-21.1)	6.1 (3.7-10.2)	6.5 (4.0-10.7)
Ratio of Mean PDMP queries, baseline to intervention (95% CI)	1.44 (1.36-1.51)		1.06 (0.99-1.13)	
Ratio between PDMP integration and control of the change from baseline (95% CI)	1.35 (1.25-1.47)			
	Clinicians with Below-Median Tenure in Medicine (<13 years)			
Monthly PDMP queries, mean for typical PCP(95% CI)	9.0 (6.1-18.7)	17.8 (12.0-26.3)	6.8 (4.5-10.1)	7.1 (4.8-10.5)
Ratio of Mean PDMP queries, baseline to intervention (95% CI)	1.98 (1.87-2.11)		1.04 (0.99-1.11)	
Ratio between PDMP integration and control of the change from baseline (95% CI)	1.89 (1.73-2.05)			

© 2022 Neprash HT et al. *JAMA Health Forum.*

eTable 4. Changes in Adjusted PDMP Query Frequency, across 4 Treatment Arms

Characteristic	PDMP Integration		PDMP Integration + Choice Architecture Intervention		Choice Architecture Intervention		Control Group	
	Baseline Period	Intervention Period	Baseline Period	Intervention Period	Baseline Period	Intervention Period	Baseline Period	Intervention Period
Monthly PDMP queries, mean for typical PCP(95% CI)	12.2 (7.2-20.5)	21.2 (12.5-35.8)	6.6 (4.1-10.8)	10.7 (6.5-17.5)	10.7 (6.4-17.8)	11.9 (7.1-19.8)	4.1 (2.4-6.8)	4.1 (2.4-6.8)
Ratio of Mean PDMP queries, baseline to intervention (95% CI)	1.74 (1.65-1.83)		1.61 (1.52-1.70)		1.11 (1.04-1.17)		1.00 (0.94-1.06)	
Ratio between treatment arm and control of the change from baseline (95% CI)	1.74 (1.61-1.89)		1.61 (1.48-1.75)		1.11 (1.02-1.21)		-	

© 2022 Neprash HT et al. *JAMA Health Forum.*

eFigure. Clinician-Level Count of PDMP Queries/Month, across 4 Treatment Arms

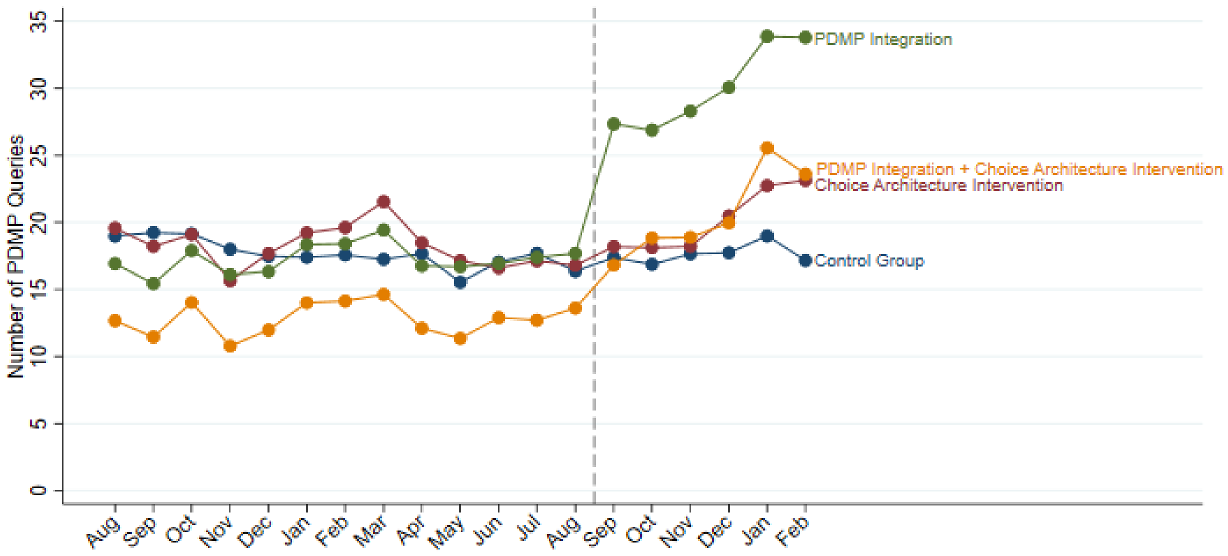

Source: Authors' analysis of Minnesota Board of Pharmacy Data.
Notes: PDMP is prescription drug monitoring program.

© 2022 Neprash HT et al. *JAMA Health Forum.*
