# Supplementary material for: Effect of Integrating Access to a Prescription Drug Monitoring Program Within the Electronic Health Record on the Frequency of Queries by Primary Care Clinicians: A Cluster Randomized Clinical Trial
Source: JAMA Health Forum. 2022 Jun 5;3(6):e221852. doi: 10.1001/jamahealthforum.2022.1852 (PMC9168784; doi:10.1001/jamahealthforum.2022.1852)
Supplement: Supplement 3. — Data Sharing Statement [file jamahealthforum-e221852-s00.pdf]

Data Sharing Statement

Neprash. Effect of Integrating Access to a Prescription Drug Monitoring Program Within the Electronic Health Record on the Frequency of Queries by Primary Care Clinicians. *JAMA Health Forum*. Published June 05, 2022. doi:10.1001/jamahealthforum.2022.1852

Data
Data available: No

Additional Information
Explanation for why data not available: Data are only available to PRINCE study researchers via a data use agreement with the Minnesota Board of Pharmacy
